# The Impact of Volume Removal by Hemodialysis on Elevated NT-proBNP Levels in Patients With Hypervolemia

**DOI:** 10.7759/cureus.85554

**Published:** 2025-06-08

**Authors:** Mohammad Tinawi, Bahar Bastani

**Affiliations:** 1 Nephrology, Nephrology Specialists, Munster, USA; 2 Nephrology, Saint Louis University School of Medicine, Saint Louis, USA

**Keywords:** acute kidney injury (aki), brain natriuretic peptide (bnp), congestive heart failure (chf), end-stage renal disease (esrd), hemodialysis (hd), nt pro-bnp

## Abstract

Introduction and aim: N-terminal pro-brain-type natriuretic peptide (NT-proBNP) is highly sensitive and specific for the diagnosis of congestive heart failure (CHF). Most patients with end-stage kidney disease (ESKD) on renal replacement therapy have an elevated NT-proBNP plasma level due to volume gain between dialysis sessions. Measuring plasma NT-proBNP levels before and after hemodialysis (HD) in patients hospitalized with volume expansion is a common practice. This study aimed to determine the clinical value of repeated plasma NT-proBNP measurements in patients with ESKD or acute kidney injury (AKI) requiring HD and to study the effect of volume removal via HD on NT-proBNP plasma levels.

Methods: We conducted a retrospective analysis of patients admitted to two community hospitals over a three-year period (from January 1, 2020, to December 31, 2022). Patients with AKI requiring HD or ESKD were screened if they had an admission diagnosis of volume expansion including acute CHF or pulmonary edema that was verified radiographically and by physical examination. Patients who had a plasma NT-proBNP level measured at admission and a repeat measurement 2-12 hours after HD were included in this study. Over the three-year period, 36 patients with ESKD and five patients with AKI requiring HD fulfilled the inclusion criteria.

Results: Volume removal via HD had no impact on NT-proBNP levels in ESKD or AKI patients (ESKD p=0.7858 and AKI p=0.6903). Our patients exhibited markedly elevated NT-proBNP due to volume expansion and renal dysfunction. During the study period, 30% of the patients with ESKD (11/36) died.

Conclusion: Volume expansion is the main factor causing elevated NT-proBNP levels in acute or chronic HD patients. Reducing predialysis volume expansion with a single hemodialysis session has no impact on NT-proBNP level. Therefore, repeating the NT-proBNP level post-HD has no clinical value. Our data suggest that NT-proBNP is a useful cardiac biomarker for volume expansion, and its elevated level predicts high cardiovascular mortality and recurrent hospitalizations in acute and chronic HD patients.

## Introduction

PreproBNP is a 134 amino acid peptide synthesized in the cardiac myocytes. After processing, it becomes pro-brain-type natriuretic peptide (proBNP), a vasopeptide prohormone consisting of 108 amino acids. ProBNP is synthesized and secreted mainly by the myocardium in response to increased ventricular wall stretch, as in volume expansion [1]. It is cleaved in the circulation into two peptides. The 3.5 kDa 32-amino acid C-terminal is the active fragment and is a natriuretic hormone known as brain-type natriuretic peptide (BNP). It was originally isolated from the brain in 1988. BNP has a diuretic, natriuretic, and vasodilatory effect. Additionally, it inhibits the production of renin and aldosterone. The human gene encoding BNP was mapped to chromosome 1. Cardiac fibrosis is seen in BNP gene knockout mice, while BNP gene overexpression in mice leads to bone malformation and hypotension. The role of the BNP gene in humans requires further elucidation.

The inert fragment is the 8.5 kDa 76-amino acid N-terminal known as NT-proBNP. Both peptides are widely used for the diagnosis of congestive heart failure (CHF) [2]. In the N-terminal proBNP Investigation of Dyspnea in the Emergency Department (PRIDE) Study, an NT-proBNP value <300 pg/mL ruled out acute CHF with a 99% negative predictive value [3]. For patients <50 years of age, a cut-off value of >450 pg/mL had a sensitivity of 98% and specificity of 93% for diagnosing CHF. The cut-off value that achieves the same sensitivity and specificity for patients 50 years of age was >900 pg/mL.

The level of NT-proBNP is higher than the level of BNP in patients with CHF. BNP is cleared from plasma via proteolysis by neutral endopeptidases or by binding to the natriuretic peptide receptor type C. Renal elimination of BNP is minor, but the exact contribution of the kidney to BNP clearance is unclear [4]. NT-proBNP is cleared by renal excretion, which explains the strong effect of kidney function on its concentration. It is unclear whether specific renal metabolic pathways exist. Elimination in patients with low urine output is negligible. The diagnostic cutoffs for NT-proBNP increase with age due to the decrease in renal function associated with aging [5]. Approximately half of asymptomatic stage 3 and 4 chronic kidney disease patients have elevated NT-proBNP levels, median value of 490 pg/mL (range: 122-1,898 pg/mL) [6]. Almost all end-stage kidney disease (ESKD) patients on hemodialysis (HD) or peritoneal dialysis have elevated NT-proBNP levels, but there is no agreement on the cutoffs [4,7,8]. This elevation in ESKD patients is attributed to the renal clearance of NT-proBNP, and the high prevalence of volume expansion and cardiac abnormalities such as left ventricular hypertrophy and CHF [4,7]. NT-proBNP has gained wide clinical acceptance because the assays are standardized, which makes testing variability less pronounced compared to BNP [9]. The half-life of NT-proBNP is 60-120 minutes, which is longer than the BNP half-life of 20 minutes [10].

Measurement of plasma NT-proBNP upon hospital admission in HD patients with volume expansion is a common practice. Many clinicians repeat the measurement after volume removal with HD to determine if the NT-proBNP level has declined [4,7,9]. The precise role of NT-proBNP measurement in dialysis patients requires further research. This retrospective analysis aimed to determine the clinical value of NT-proBNP measurements pre- and post-HD in patients with clinical volume overload who are ESKD on chronic intermittent HD, ESKD initiating HD, or acute kidney injury (AKI) requiring HD. We also sought to study the effect of volume removal with high-flux HD on plasma NT-proBNP levels.

## Materials and methods

**Figure 1:**
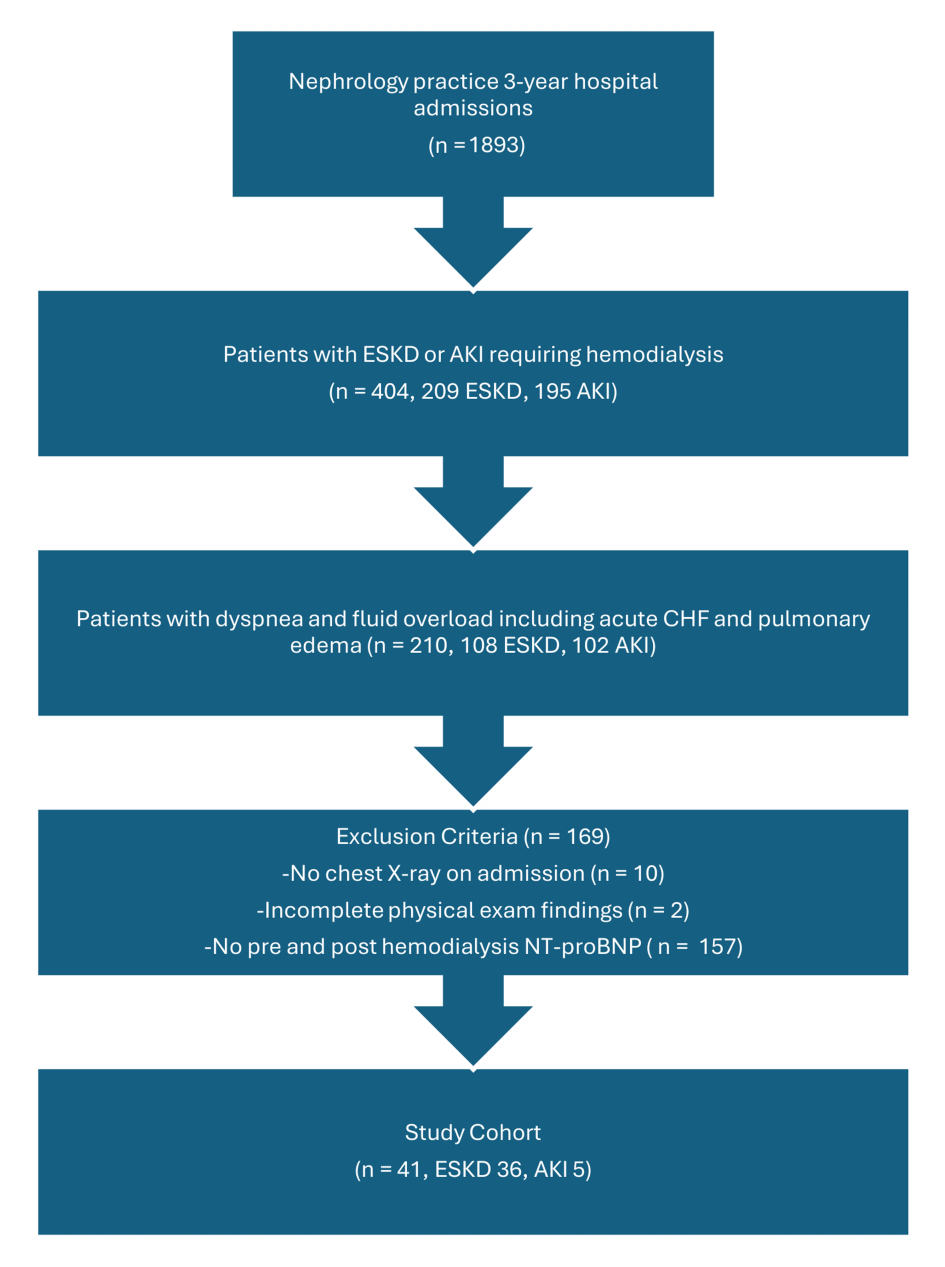
Population flow chart. ESKD: end-stage kidney disease; AKI: acute kidney injury; NT-proBNP: N-terminal pro-brain-type natriuretic peptide; CHF: congestive heart failure

We conducted a retrospective analysis of patients admitted to two community hospitals under the care of a single-specialty nephrology practice over a three-year period (from January 1, 2020, to December 31, 2022). This study aimed to evaluate hemodialysis efficacy in reducing volume overload (evaluated based on fluid overload indicators), as reflected by NT-proBNP levels, in patients with AKI or ESKD admitted for acute dyspnea. Inclusion criteria were patients admitted with a diagnosis of AKI or ESKD, irrespective of duration on HD. All patients had to be admitted with dyspnea and clinical evidence of volume expansion including acute CHF or frank pulmonary edema. All patients had a chest X-ray on admission demonstrating increased vascular congestion or frank pulmonary edema. Physical examination should have been compatible with a significant volume expansion, i.e., the presence of crackles on chest examination, and pitting edema in the lower extremities. All patients were either already on intermittent HD in case of established ESKD, were started on HD upon admission due to a new diagnosis of ESKD, or required HD due to AKI. All patients required volume removal with their inpatient HD. Patients had to have a plasma NT-proBNP level drawn upon admission <24 hours before their first inpatient HD session, and a repeat level 2-12 hours after the same HD session. Although a 2-12 hours window is broad, it was deemed acceptable based on the long half-life of NT-proBNP, but this issue requires further research. Patients were excluded if they did not have a chest X-ray on admission, if physical examination findings were incomplete, or if NT-proBNP was not measured pre- and post-HD. Over the three-year period, 108 ESKD patients and 102 AKI patients were admitted with dyspnea and fluid overload, of whom 36 patients with ESKD and five patients with AKI fulfilled the inclusion criteria (Figure 1).

All patients received HD using Fresenius 2008T machines (Bad Homburg, Germany: Fresenius Medical Care). HD was done with single-use high-flux dialyzers Optiflux 160 (Bad Homburg, Germany: Fresenius Medical Care) (surface area 1.5 m^2^, ultrafiltration coefficient 61), or 180 (surface area 1.7 m^2^, ultrafiltration coefficient 76). Removal of BNP and NT-proBNP (both are mid-molecular weight molecules) by high-flux dialyzers is variable but minor. This is discussed further below. The difference in surface area between the two dialyzers is only 0.2 m^2^, and the data were not stratified based on type of dialyzer.

NT-proBNP was analyzed using electrochemiluminescence immunoassay (ECLIA) Elecsys proBNP II (Basel, Switzerland: Roche Diagnostics). The NT-proBNP measuring range was 5‐35,000 pg/mL; values below the lower detection limit were reported as <5 pg/mL. Values above the measuring range were reported as >35,000 pg/mL. The coefficient of variation was 20% or <50 pg/mL. Samples with NT‐proBNP concentrations above the measuring range could be diluted with Diluent Universal.

For the purpose of statistical analysis, NT-proBNP values >35,000 pg/mL were considered 35,000 pg/mL. NT-proBNP values are expressed as mean±standard deviation (SD). Certain values are reported as median with range or quartiles in parentheses. Differences between groups were analyzed with Student’s t-tests. P<0.05 was considered statistically significant. The data were also analyzed using the Wilcoxon signed-rank test.

## Results

The demographics and baseline characteristics of the study patients are shown in Table 1. The main findings of this study are summarized in Table 2. The data for the ESKD and AKI groups are reported side-by-side; however, direct comparison is not possible due to the large disparity in the size of the two groups. Residual renal function was not assessed.

**Table 1:** The baseline demographics and characteristics of the study patients. Regarding time on HD, the value (0) signifies a diagnosis of ESKD made on admission. ESKD: end-stage kidney disease; AKI: acute kidney injury; W: White; B: African American; H: Hispanic; HD: hemodialysis; CHF: congestive heart failure

Variables	ESKD, n=36 patients	AKI, n=5 patients
Age (years), mean (range)	63 (37-86)	62 (51-76)
Sex		
Male, n	19	3
Female, n	17	2
Race		
W, n (%)	9 (25%)	1 (20%)
B, n (%)	19 (53%)	3 (60%)
H, n (%)	8 (22%)	1 (20%)
Time on HD in months	13 (0-151)	N/A
Mean (range)		
History of CHF, n (%)	8 (22%)	2 (40%)
History of hypertension, n (%)	28 (78%)	2 (40%)
History of diabetes mellitus, n (%)	9 (25%)	2 (40%)
Pulmonary edema on presentation, n (%)	12 (33%)	0

**Table 2:** The main findings of the study. NT-proBNP reference ranges for the diagnosis of CHF according to age: <50 years of age = 450 pg/mL; 50-75 years of age >900 pg/mL; and >75 years of age >1,800 pg/mL. ESKD: end-stage kidney disease; AKI: acute kidney injury; NT-proBNP: N-terminal pro-brain-type natriuretic peptide

Variables	ESKD, n=36	AKI, n=5
Duration of HD session in hours, mean (range)	3.3 (2.5-4)	2.8 (2.5-3)
Volume removal in litres, mean (range)	2.5 (1-5)	1.3 (1-2)
Urea reduction ratio, mean±SD (range)	64.1±8%	63.2±7%
Number of hospitalizations after the initial presentation during the study period, median (range)	3.0 (0-14)	1 (0-3)
Mortality over the study period, n (%)	11 (30%)	3 (60%)
Time from initial presentation until death in months, median (range)	6 (1-21)	1 (1-13)
Pre-HD NT-proBNP (pg/mL), mean±SD (range)	29,151±10,039 (4,113-35,000)	25,823±12,271 (4,270-35,000)
Post-HD NT-proBNP (pg/mL), mean±SD (range)	28,980±9,815 (4,440-35,000)	26,523±11,339 (4,736-35,000)

Of patients with ESKD, 23 out of 36 (64%) had pre-HD NT-proBNP levels reported as >35,000 pg/mL, and these levels remained as >35,000 pg/mL post-HD. The same applied to two out of five (40%) of patients with AKI. The upper calibrated range for the assay used was 35,000 pg/mL. Values above that range were reported as >35,000 pg/mL. Determination of the exact values requires further dilutions that were not done by the laboratory, and given the retrospective nature of this study, it cannot be determined. It is conceivable that post-HD NT-proBNP levels would be lower in some of these patients, but given the very elevated pre- and post-HD NT-proBNP levels in these patients (>35,000 pg/mL), an exact determination is not likely to be of clinical significance; however, this requires further research. Urea reduction ratio was generally adequate in both ESKD and AKI patients (Table 2).

Although volume expansion is the main factor causing elevated NT-proBNP levels in acute or chronic HD patients, our study shows that correction of predialysis volume expansion with a single HD session had no impact on NT-proBNP levels within the laboratory test calibration range in patients with ESKD or AKI. Our patients exhibited markedly elevated levels due to their renal dysfunction and the presence of volume expansion on presentation. In patients with ESKD, the pre-HD NT-proBNP level (mean±SD) was 29,151±10,039 pg/mL, and the post-HD NT-proBNP level (mean±SD) was 28,980±9,815 pg/mL. The mean±SD decline in NT-proBNP levels post-HD was 298±3,666 pg/mL (p=0.7858). The very large standard deviation is because post-HD NT-proBNP levels did not change in 64% (23/36 patients), were higher in 25% (9/36 patients) (mean increase±SD: 2371.6±2,448 pg/mL), and were lower in only 11% (4/36 patients), (mean decline±SD: 6,878±8,297 pg/mL). The ESRD data were then analyzed using Wilcoxon signed-rank test. For a two-tailed test and alpha=0.05, the mean±SD=430±244, Z-score -1.03, the critical Z-values are ±1.96, since Z-score is within that range, the result is not significant at the 5% level, which is the same conclusion reached with Student's t-test. Of the 11 ESKD patients (30%) who died during the study period, eight (73%) had pre- and post-HD NT-proBNP levels >35,000 pg/mL, and five (45%) died of cardiovascular causes.

All AKI patients had acute tubular necrosis. The findings were similar in five patients with AKI. Two patients died of cardiovascular causes during the study period. The mean pre-HD NT-proBNP level was 25,823±12,271 pg/mL, and the mean post-HD level was 26,523±11,339 pg/mL. The mean increase in post-HD NT-proBNP levels was 700±3,266 pg/mL, p=0.6903. The AKI data were then analyzed using Wilcoxon signed-rank test. For a two-tailed test and alpha=0.05, the mean±SD was 7.5±3.7, Z-score was -0.944, the critical Z-values were ±1.96, since Z-score was within that range, the result was not significant at the 5% level, which was the same conclusion reached with Student's t-test. The small sample size in the AKI group requires interpretation of the results with caution.

**Figure 2:**
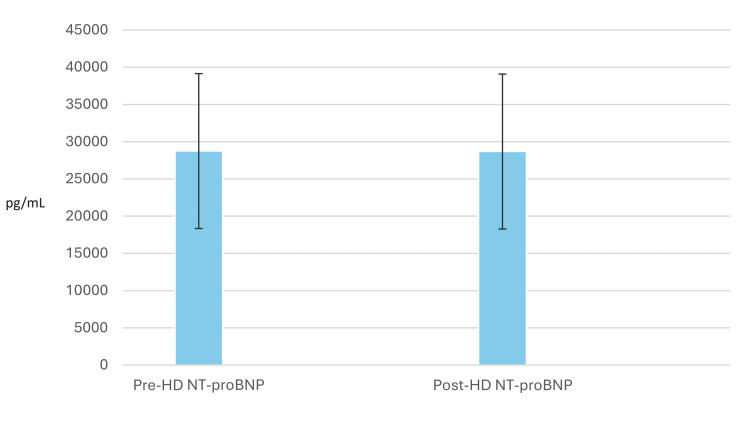
Bar diagram showing lack of change in NT-proBNP levels post-hemodialysis, the data were combined for end-stage kidney disease and acute kidney injury patients. NT-proBNP: N-terminal pro-brain-type natriuretic peptide

Combining the data from ESKD and AKI patients yielded the same results. The mean pre-HD NT-proBNP level was 28,745±10,403 pg/mL, and the mean post-HD level was 28,680±10,051 pg/mL. The mean decline in post-HD NT-proBNP levels was 65±3,708 pg/mL, p=0.9110 (Figure 2). Several confounding factors should be considered including left ventricular ejection fraction, the dose of dialysis (Kt/V), and ultrafiltration volume.

## Discussion

NT-proBNP is a useful test in patients with chronic kidney disease who present with dyspnea to diagnose acute CHF. Data analysis from the PRIDE study showed that NT-proBNP helped exclude CHF irrespective of renal function (negative predictive value 94% and 100% for patients with glomerular filtration rate <60 mL/min/1.73 m^2^ and ≥60 mL/min/1.73 m^2^, respectively), the cut-off value was 300 pg/mL [11]. NT-proBNP sensitivity and specificity for identifying CHF were 97% and 68%, respectively, for patients with glomerular filtration rate <60 mL/min/1.73 m^2^, irrespective of age. For patients with glomerular filtration rate >15 and <44 mL/min/1.73 m^2^, a cut-off value of 1,200 pg/mL has a 92% sensitivity and 70% specificity for CHF diagnosis.

Chazot et al. analyzed the records of 236 HD patients for six months starting at HD initiation [12]. At HD initiation, the median BNP was 593 pg/mL (range: 175-1,433 pg/mL). The approximately 50% decrease in BNP level between month one and month two correlated with fluid removal. After the second month, the BNP level plateaued. Median BNP was 291 pg/mL in non-CHF patients, and 2.5 times higher (731 pg/mL) in CHF patients. Patients with the highest BNP tertile (>1,070 pg/mL) had the highest mortality.

The usefulness of BNP and NT-proBNP as markers of volume expansion in HD patients is a matter of debate. In a study of 150 hypertensive HD patients, Agarwal concluded that BNP is not useful as a marker of volume expansion or as a tool for the assessment of dry weight [13]. Wang et al. studied 129 chronic HD patients and concluded that NT-proBNP is a useful predictor of volume expansion in HD patients irrespective of left ventricular ejection fraction [14]. The investigators performed NT-proBNP measurements, bioimpedance spectroscopy, and transthoracic echocardiogram (TEE) on a midweek non-dialysis day. The median NT-proBNP level was 4,992 (range: 2,033-15,807) pg/mL. In HD patients with left ventricular ejection fraction <60%, the NT-proBNP cutoff value was 15,617 pg/mL with a sensitivity of 92% and a specificity of 73% for predicting volume expansion.

Our study shows that volume removal via a single HD session does not affect the already elevated NT-proBNP levels in patients with volume expansion and ESKD or AKI. Thus, the common practice of repeating NT-proBNP level soon after volume removal with a single HD session has no clinical value. The long half-life of NT-proBNP (60-120 minutes) is a factor [14]. Removal of 1-5 L over the course of one HD session did not significantly affect elevated NT-proBNP levels. However, it is conceivable that aggressive fluid removal over several weeks may result in NT-proBNP level reduction. It is unclear if dialysis vintage had an effect on NT-proBNP levels.

NT-proBNP level is elevated in ESKD patients; however, different studies have provided different cutoffs. In our study, NT-proBNP levels were significantly elevated because only patients with ESKD or AKI and clear evidence of volume expansion were included in the analysis. In a study of 134 HD patients, Sommerer et al. reported that all HD patients studied had significantly elevated levels of NT-proBNP (median: 4,524, interquartile range: 2,000-10,250 pg/mL) [15]. The levels were significantly higher in hypervolemic HD patients (median: 11,988, interquartile range: 5,307-19,242 pg/mL), p<0.001. They concluded that NT-proBNP is highly predictive of hypervolemia in HD patients.

David et al. assessed left ventricular function in 62 stable chronic HD patients via TEE [16]. The investigators performed bioelectrical impedance analysis (BIA) to assess extracellular water. They concluded that a serum NT-proBNP cut-off value of 7,200 pg/mL can identify HD patients with left ventricular dysfunction with a sensitivity of 79% and a specificity of 90%, irrespective of volume status. In those patients, elevated NT-proBNP levels correlate with persistent post-HD volume expansion.

Our data do not show a significant effect of high-flux membrane HD on NT-proBNP levels. This is likely due to the significant elevation in NT-proBNP level pre-dialysis (mean±SD: 29,151±10,039 pg/mL) in patients with clear volume expansion, which makes the effect of high-flux membrane HD negligible. Wahl et al. studied the effect of HD on NT-proBNP levels in 17 patients. NT-proBNP was elevated in all patients pre-dialysis (mean±SE: 25,366±9,062 ng/L). Dialysis with a high-flux membrane led to a minor decrease of 2.3% in NT-proBNP level (13 patients), while the level increased by 4.9% in the four patients dialyzed with a low-flux membrane. The cause of this discrepancy could be related to the 8.5 kDa molecular mass of NT-proBNP, which precludes clearance by low-flux membranes. The increase in post-dialysis level was explained by the apparent release of NT-proBNP into the circulation during HD, according to the authors. The patients’ characteristics and timing of specimen collection were not provided [5].

Madsen et al. measured serum NT-proBNP pre- and post-HD in 109 patients. NT-proBNP was significantly elevated pre-HD 4,079 pg/mL (median: interquartile range 1,893-15,076), and post-HD 2,759 pg/mL (1,078-11,070 pg/mL), p<0.001 [7]. Median pre-HD NT-proBNP levels were about five times higher in CHF patients compared to those with no CHF diagnosis. The mean reduction in NT-proBNP levels post-HD was 38.8±13.6%. The amount of fluid removal during HD had no effect on the post-HD NT-proBNP levels. NT-proBNP was drawn just before and at the end of HD. All patients were dialyzed for 3-4.5 h using a high-flux dialyzer. Post-HD NT-proBNP levels were lower than pre-HD levels, and both were predictive of death. NT-proBNP correlated positively with left ventricular hypertrophy, and negatively with left ventricular ejection fraction, 24-h urine output, and dialysis adequacy (Kt/V). We did not see a reduction in NT-proBNP post-dialysis in our patient, presumably due to the significantly elevated pre-HD levels. Our data show that NT-proBNP is not useful for dry (target) weight determination due to the lack of correlation between NT-proBNP levels and volume removal with HD. Others have reached a similar conclusion [7,17].

In our analysis, 30% of ESKD patients (11/36) died over the three-year study period. The relationship between elevated NT-proBNP levels in ESKD patients and mortality is well established. Our ESKD patients had a median of three hospitalizations post the initial encounter during the study period. Satyan et al. concluded that NT-proBNP level had a strong graded relationship with cardiovascular and all-cause mortality in 150 asymptomatic HD patients after a median follow-up of 24 months. The relationship persisted after adjusting for mid-wall fractional shortening (a marker of systolic dysfunction in patients with left ventricular hypertrophy) and left ventricular mass index [8]. In a study of 246 HD and PD patients without CHF, Zoccali et al. found that BNP level is a predictor of ejection fraction, overall and cardiovascular mortality [18]. Sato et al. followed 920 chronic HD patients for up to 84 months and found that NT-proBNP levels are significantly higher in females, elderly, and low dry weight patients. NT-proBNP was significantly associated with cardiovascular and all-cause mortality [19].

A study by Sharma et al. in 144 kidney transplant candidates on HD and peritoneal dialysis concluded that NT-proBNP is a marker of mortality, coronary artery disease, and left ventricular dysfunction in ESKD [20]. Other investigators have reached a similar conclusion [7,8,15,18,21-23]. The prognostic value of NT-proBNP persisted irrespective of the timing of the specimen collection, pre-dialysis, post-dialysis, or intra-dialysis.

Gutiérrez et al. concluded that NT-proBNP levels are independently associated with mortality in incident HD patients [24]. Moreover, serial measurements of NT-proBNP levels were valuable in predicting mortality in incident cases. Patients with the highest increase in NT-proBNP levels after three months of HD had an approximately 2.5 times higher mortality risk compared to patients with the greatest decrease. In a meta-analysis of 49 studies, Harrison et al. found a relationship between NT-proBNP and BNP levels in ESKD patients and cardiovascular and all-cause mortality [23]. All-cause mortality HR was 2.31 (95% CI: 1.48-3.61) for NT-proBNP >2,000 pg/mL, and 3.92 (95% CI: 1.42-10.82) for NT-proBNP >20,000 pg/mL.

Our analysis included five patients with AKI. The results were analogous to those observed in patients with ESKD. There is a paucity of data regarding NT-proBNP in patients with AKI and volume expansion. Its diagnostic and prognostic value is unclear and requires further study. de Cal et al. conducted a study in 34 consecutive intensive care patients and concluded that high levels of plasma BNP can help in detecting patients with a high risk of AKI in the ICU setting [25].

Our study has several limitations. It is retrospective and observational in nature. It lacks a control group. Long-term outcome data beyond the study period are not available. Verification of volume expansion based on radiographic and physical examination findings can be subjective, and our study did not use direct volume measurement such as bioimpedance. Certain parameters were not included in the analysis, such as blood and dialysate flow rates, and anticoagulation use. These variables may impact volume removal and NT-proBNP dynamics. NT-proBNP levels were measured only twice in each patient (pre- and post-HD). Residual kidney function was not measured. NT-proBNP was not measured in the dialysate. The study design cannot provide predictions regarding morbidity and mortality.

## Conclusions

Our study shows that the common practice of measuring NT-proBNP level after a single HD session to document a positive effect of volume removal is of no value. Rather, identifying HD patients with significant NT-proBNP elevation to intensify their cardiovascular management may be beneficial. HD, even with a high-flux membrane, does not have a significant effect on elevated NT-proBNP levels in patients presenting with fluid overload. A longitudinal follow-up over several weeks and months is worth considering. The frequency and timing of specimen collection need further study and standardization. This study is a retrospective analysis and, therefore, hypothesis-generating. A cause and effect cannot be defined. Prospective studies with serial NT-proBNP measurements over time and correlation with volume status and outcome are needed. Our data suggest that NT-proBNP is a useful cardiac biomarker for volume expansion and in predicting cardiovascular mortality and recurrent hospitalizations. Finally, the role of NT-proBNP in AKI patients requiring HD needs further elucidation.
